# Recurrence quantification analysis of postural sway in patients with persistent postural perceptual dizziness

**DOI:** 10.3389/fresc.2023.1142018

**Published:** 2023-07-27

**Authors:** Megan J. Kobel, Andrew R. Wagner, Daniel M. Merfeld

**Affiliations:** ^1^ Department of Otolaryngology—Head & Neck Surgery, Ohio State University Wexner Medical Center, Columbus, OH, United States; ^2^ Department of Health & Rehabilitation Sciences, Ohio State University, Columbus, OH, United States; ^3^ Department of Speech and Hearing Science, Ohio State University, Columbus, OH, United States; ^4^ Department of Biomedical Engineering, Ohio State University, Columbus, OH, United States

**Keywords:** persistent postural perceptual dizziness, recurrent quantification analysis, postural balance, vestibular, postural control

## Abstract

**Background:**

Persistent postural perceptual dizziness (PPPD) is a common cause of chronic dizziness and imbalance. Emerging evidence suggests that changes in quantitative measures of postural control may help identify individuals with PPPD, however, traditional linear metrics of sway have yielded inconsistent results. Methodologies to examine the temporal structure of sway, including recurrent quantification analysis (RQA), have identified unique changes in dynamic structure of postural control in other patient populations. This study aimed to determine if adults with PPPD exhibit changes in the dynamic structure of sway and whether this change is modulated on the basis of available sensory cues.

**Methods:**

Twelve adults diagnosed with PPPD and twelve age-matched controls, completed a standard battery of quiet stance balance tasks that involved the manipulation of visual and/or proprioceptive feedback. For each group, the regularity and complexity of the CoP signal was assessed using RQA and the magnitude and variability of the CoP signal was quantified using traditional linear measures.

**Results:**

An overall effect of participant group (i.e., healthy controls vs. PPPD) was seen for non-linear measures of temporal complexity quantified using RQA. Changes in determinism (i.e., regularity) were also modulated on the basis of availability of sensory cues in patients with PPPD. No between-group difference was identified for linear measures assessing amount and variability of sway.

**Conclusions:**

Participants with PPPD on average exhibited sway that was similar in magnitude to, but significantly more repeatable and less complex than, healthy controls. These data show that non-linear measures provide unique information regarding the effect of PPPD on postural control, and as a result, may serve as potential rehabilitation outcome measures.

## 1. Introduction

Persistent postural perceptual dizziness (PPPD) is a chronic, functional vestibular disorder characterized by persistent non-spinning vertigo, dizziness, and imbalance exacerbated by active or passive self-motion, and exposure to complex visual stimulation (1). PPPD is one of the most common diagnoses in patients with chronic dizziness (2, 3) and onset regularly occurs following a vestibular or alternative medical event that yields dizziness and/or imbalance (1, 4, 5). A hallmark of PPPD is perceived chronic postural instability (1) and analysis of postural control and postural sway have been suggested to potentially play a role in identifying PPPD (6). However, the mechanisms underlying PPPD are not fully known, and the potential utility of postural sway in identifying PPPD is incompletely characterized.

The diagnostic criteria for PPPD have only recently been established (1). Thus, hypotheses pertaining to the mechanisms underlying PPPD and perceived instability must also be viewed in the context of prior investigations of individuals with past diagnoses such as phobic postural vertigo (PPV) and chronic subjective dizziness (CSD). Previous investigations in patients diagnosed with PPV have revealed an increase in sway at high frequencies (7) and an increase in the velocity of sway in patients with CSD (8). Similarly, recent studies in patients with PPPD identified increased low frequency and decreased high frequency sway (9). Such findings are consistent with a stiffened strategy for postural control (i.e., co-contraction of lower limb musculature). These findings, in conjunction with the characteristic reports of perceived postural instability, support the supposition that individuals with PPPD adopt a maladaptive high-risk, stiffened postural control strategy (1, 5). While these adaptive strategies are beneficial in the acute phase of vestibular disorders, a failure to re-adapt the postural control strategy in patients with PPPD suggests that these strategies may be influenced by inadequate higher level cortical control and attentional hypervigilance (1, 10). In PPV, an attentional component to postural control is supported by past findings demonstrating reductions in amount of postural sway and reduction in postural stiffness during dual task performance (11) and increased balance performance (i.e., decreased postural sway) during more complex balance tasks (12).

In balance assessments, center of pressure (CoP) motion is commonly quantified and traditional (i.e., linear) measures of CoP have focused on quantifying the behavior of the CoP in the time domain (13–16). However, these measures fail to capture dynamic aspects of the CoP (i.e., how CoP motion changes over time) and assume stationarity of the signal. Recent evidence suggests that non-linear analytic techniques characterizing the dynamic temporal structure of postural sway may provide unique insights into functional organization of the postural control system [e.g., (14, 16, 17)]. These non-linear measures of CoP dynamics, including recurrent quantification analysis (RQA), provide an alternative to traditional posturographic assessments and have been proposed to more reliably quantify sway than traditional amplitude-based metrics (18). Additionally, several studies have identified changes in postural control strategy not captured by linear measures (14, 17, 19, 20) including individuals with imbalance from musculoskeletal pain, stroke, mild traumatic brain injury, and Parkinson's Disease (21–26). However, applications quantifying nonlinear sway metrics in PPPD patients are limited.

While there are several methods to characterize patterns of the dynamic CoP signal including RQA, detrended fluctuation analysis (DFA), sample entropy, and stabilogram diffusion analysis (SDA), methodological constraints exist in the context of non-stationary data and bounded time series of CoP trajectories when implementing non-linear techniques such as SDA (27, 28). Due to these constraints, several authors have proposed implementation of RQA for CoP time series, since this methodology has been extensively investigated by other fields (e.g., mathematics) and is linked to well defined concepts from statistical physics and nonlinear dynamics (16, 17, 25).

**Figure 1:**
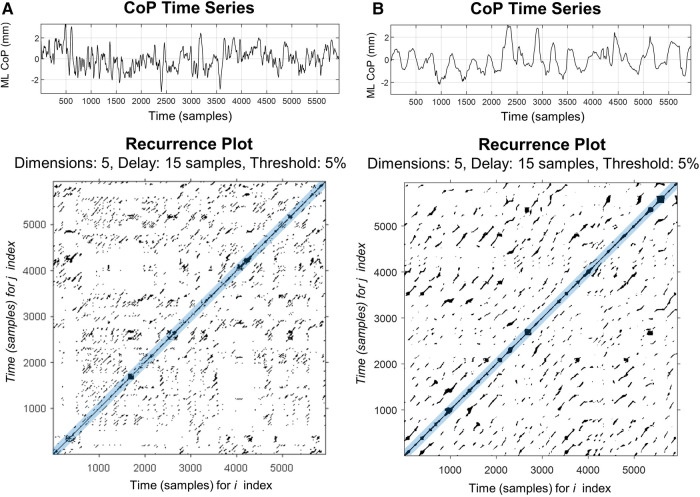
Representative recurrence plots of mediolateral CoP motion for a healthy control (**A**) and patients with PPPD (**B**) during a quiet stance on a firm surface with eyes open (condition 1). Plots were made using Marwan RQA Toolbox (v.5.24 (R34) (34). In the upper panels, the 60 s CoP time series, sampled at 100 Hz, is plotted for both participants. In the lower panels, the two-dimensional recurrence plots of the same time series are shown; these represent comparisons between two time-lagged CoP signals in multi-dimensional space. Darkened points represent points which are recurrent in time and are neighbors in the reconstructed phase space. Main diagonal (i.e., line of identify) is due to comparing each point to itself. Blue shaded region around the main diagonal represents the Theiler window, which excludes temporally close recurrence from data analysis.

RQA was developed as a quantitative extension of the recurrence plot (RP), permitting a numerical, as opposed to a visual, description of the underlying dynamic behavior of a scalar times series. A full overview of RQA and RPs are outside the scope of this paper, however, several in-depth tutorials exist (14, 29–31). In brief, RPs provide a way to visualize the behavior of a higher dimensional dynamic system (32) as the RP is simply a graphical depiction of a recurrence matrix rooted in phase space reconstruction (14). Recurrence is determined by first reconstructing the original CoP signal in phase space by creating several (*m*) time delayed vectors of the original CoP time series. Each vector is delayed by a multiple of the time delay (*τ*) such that X(i)=x(i),x(i+τ),x(i+2τ),…(x(i+(m−1)τ)] (33). The distances between all possible vectors in the reconstructed phase space are determined and used to generate a distance matrix. Recurrent points are considered those points in the distance matrix that fall within a specified distance (*r*) of one another, thus, are considered to be in the same mathematical neighborhood. The RP depicts the recurrence matrix (*R_i_*_,*j*_) graphically in which when the *m*-dimensional point *x* (*i*) is in the mathematical neighborhood of *x*(*j*) (i.e., is recurrent), the location (*i*, *j*) is signified by a darkened region on the RP (Figure 1).

RQA is a quantitative extension of the qualitative RP used to describe the predictability, complexity, and regularity of the CoP time-series signal. RQA provides a means to numerically describe the patterns visualized within the recurrence plot, focusing primarily upon the diagonal lines (Figure 1). Diagonal lines within the recurrence plot represent the local evolution of unique parts of the underlying trajectory. For a completely random signal, few diagonals will be present and conversely, for a predictable time series (e.g., sine wave), long diagonal structures will be observed (14, 30, 34). These diagonal structures have been shown to be related to the predictability of the signal (34, 35), and thus serve as a surrogate to quantitatively describe the underlying dynamics of the CoP signal. Usually, in a dynamic system such as postural sway, a complete analysis is only possible when all equations of motion and degrees of freedom are known (e.g., displacement, velocity, acceleration); however, often only a single variable is directly measured. RQA allows understanding of the dynamics of postural sway through examining multi-dimensional space, while only surveying a single behavioral variable (e.g., the mediolateral CoP displacement).

Several investigations have examined non-linear measures of postural sway and changes in CoP structure on the basis of postural control challenge and perturbation (14, 16, 17, 22, 24, 25). Postural sway, in general, has been seen to increase in both absolute amount and variability, as quantified by linear measures of sway, as balance condition becomes more difficult and as sensory information pertinent for balance is degraded or removed (14, 17). These increases in sway are accompanied by increased regularity of the temporal structure of the CoP as quantified by RQA (14, 17). Past studies have assessed CoP behavior in patients with PPV, including DFA and SDA, and demonstrated significant changes in underlying dynamics of postural control (8, 36) suggesting higher sway regularity even during less demanding balance tasks. However, these investigations were in patients with PPV, and while PPV has been superseded by PPPD, some argue for inclusion of PPV as a distinct phobic subtype of PPPD (1). As such, whether differences in postural control dynamics exist in patients meeting current PPPD diagnostic criteria has yet to be determined.

Thus, this study aimed to explore the utility of RQA to identify changes in dynamic structure of quiet stance CoP signals in patients with PPPD. In the present study, we calculated traditional linear measures and non-linear measures of the CoP trajectory using RQA in patients with PPPD and healthy controls during standard, quiet stance balance tasks designed to manipulate the reliability and/or availability of sensory cues. We hypothesized that (1) RQA metrics would reveal changes in postural control modulated by task difficulty and attentional demands not captured by linear measures, and (2) patients with PPPD would display greater regularity (i.e., inflexibility or stiffness) of the CoP signal compared to age-matched asymptomatic controls.

## 2. Methods

### 2.1. Participants

Balance performance was assessed in 12 patients diagnosed with PPPD (11F/1M; range 19–67 years, mean = 45.11, SD = 12.86) and 12 asymptomatic healthy controls (HC; 7F/5M; range 21–69 years, mean = 46.54, SD = 12.54) without a history of dizziness or vertigo. Participants in each of the two groups were age matched within two years due to the well-known decrement in balance performance that occurs with age [e.g., (13, 37)]. Patients were recruited from the oto-neurology clinic at The Ohio State University Wexner Medical Center (OSUMC) and received a diagnosis of PPPD by an oto-neurologist using ICVD criteria (38). Precipitating events for patients with PPPD included vestibular migraine (*n* = 7), COVID-19 (*n* = 1), hospitalization for an unrelated medical illness (*n* = 2), whiplash injury (*n* = 1), and panic disorder/social stress (*n* = 1). Patients who presented with PPPD in conjunction with any disorders known to be associated with permanent peripheral vestibular loss (e.g., Meniere's Disease) were excluded. In both participant groups (i.e., HC and PPPD patients), individuals were excluded on the basis of co-existing neurological disorders (e.g., stroke, multiple sclerosis), major chronic health conditions (e.g., cancer), and lower limb or musculoskeletal injuries that occurred within the previous 6 months.

All HC and PPPD participants completed standardized questionnaires assessing presence of psychiatric co-morbidities, dizziness symptom severity, and balance confidence outlined in Supplementary Table S1. All participants were screened for anxiety and depression using the Beck Anxiety Inventory [BAI; (39)] and Patient Health Questionnaire-9 [PHQ-9; (40)] but were not included/excluded on the basis of anxiety or depression. All PPPD patients reported moderate to severe anxiety on the basis of the BAI and two HC participants reported mild to moderate anxiety. Similarly, all PPPD patients reported mild to severe depression on the basis of PHQ-9 scores and one HC reported moderate depression.

Eleven patients with PPPD reported use of medications for psychiatric co-morbidities including selective serotonin reuptake inhibitors (SSRI; *n* = 6), benzodiazepine (*n* = 2), anxiolytic (*n* = 2), and atypical antipsychotic (i.e., aripiprazole; *n* = 4). Seven PPPD patients reported used of medications for migraine including anti-epileptics (i.e., topiramate; *n* = 6), beta-blockers (i.e., metoprolol; *n* = 3), or calcitonin gene-related peptide (CGRP) receptor antagonists (*n* = 4). One HC participant reported use of a SSRI, while no other HC participants reported use of medications for migraine, or psychiatric co-morbidity. Patients did not discontinue any medication prior to testing but all denied using acute medications for migraine, anxiety, or PPPD symptoms (e.g., CGRP receptor antagonists or benzodiazepines) for 2 weeks.

At the time of testing, three PPPD patients were actively enrolled in vestibular rehabilitation at OSUMC with a focus on habituation to visually provoking stimuli. All PPPD patients reported that they were still actively experiencing PPPD symptoms and indicated a moderate to severe dizziness handicap on the Dizziness Handicap Inventory (DHI) and low to moderate balance confidence on the Activities-specific Balance Confidence (ABC) Scale. No HC participants reported experiencing a significant dizziness handicap on the DHI and all reported a high balance confidence on the ABC.

The study was conducted in accordance with the Declaration of Helsinki and was approved by the Institutional Review Board of the Ohio State University (#2018H01279). All participants provided written informed consent prior to participation.

### 2.2. Balance assessment

Participants performed five balance tasks (Table 1) including each condition of the modified Romberg Test of Standing Balance (41) and an additional dual-task (i.e., counting backwards by threes) condition when standing on foam with the eyes closed. These conditions were selected to permit the assessment of balance performance in the presence of unreliable sensory information and in the presence of a secondary cognitive task. Each condition was performed once; however, if the participant did not complete a trial (e.g., uncrossed arms, opened eyes, took a step to regain balance, lost balance), the condition was repeated one additional time. Data from the final attempt was included in all data analyses. All HC participants were able to complete all conditions, while one participant with PPPD was unable to complete conditions 3 through 5 and an additional participant with PPPD could not complete conditions 4 and 5 (Table 1).

**Table 1:** Description of balance test conditions performed and sensory input available for each condition.

Condition	Vision	Surface	Sensory inputs	Participants	
				HC	PPPD
1	Eyes open	Firm	Vision, proprioception, vestibular	12	12
2	Eyes closed	Firm	Proprioception, vestibular	12	12
3	Eyes open	Foam	Vision, vestibular	12	11
4	Eyes closed	Foam	Vestibular	12	10
5	Eyes closed + dual task	Foam	Vestibular	12	10

Number of participants who were able to complete each condition and included in data analysis for each condition are presented.

For each condition, participants were asked to stand “as still as possible” with their feet positioned in narrow stance (i.e., medial border of feet touching) and their arms folded across their chest. Data was collected for 66 s, with the first 6 s removed from analysis to allow the participant to accommodate to the task. For eyes open (EO) conditions, the participants looked at a dartboard fixed at 1.524 meters (i.e., 60 in) at eye level. In the “foam” conditions, participants stood on an Airex (Somersworth, NH, US) high-density (50 kg/m^3^) closed-cell foam pad (47 cm × 39 cm × 6 cm, 0.7 kg). To mitigate any potential auditory contributions to balance performance, participants wore over the ear noise canceling headphones (Bose Quiet Comfort II) with ∼50 dB SPL of white noise presented during each balance trial.

Center of pressure (CoP) data were collected using a tri-axial force plate (AMTI, Watertown MA). CoP data were sampled at 100 Hz and prior to analysis, data were zero-meaned and low pass filtered using a 25 Hz cut off (*filtfilt*, MATLAB, Natick, MA). The primary outcome metrics were computed from CoP data collected in the mediolateral (ML) planes using custom written scripts in MATLAB (2022a). Data from the orthogonal anteroposterior (AP) plane were also captured; however, due to the exploratory nature of the study and as sway in the ML plane has been seen to correlate to falls (42, 43), we *a priori* chose to focus on metrics quantifying ML CoP. Analyses of AP sway were completed for both linear and non-linear metrics, and plane was not found to modify the effect of the fixed factors in the below-described models, and thus we report only ML metrics in the analysis in the main body. See Supplementary Tables S2, 3 for AP linear and non-linear metrics.

#### 2.2.1. Linear postural control measures

In each condition for each participant, path length and standard deviation (SD) of the CoP in the ML plane was examined. Path length is the total distance traveled by the CoP over the course of the trial duration and is approximated by calculating the sum of the distances between consecutive points on the CoP path. SD is the standard deviation of the zero-meaned CoP time series and has been shown to be related to vestibular function (44, 45). Path length and SD were selected as outcome metrics in order to mirror outcome metrics used in past studies that explicitly compared linear and non-linear measures (17). Also, patients with PPPD have previously been found to display increased sway area and increased sway variability (6, 9, 46).

#### 2.2.2. Non-linear postural control measures

RQA analysis was performed using Marwan's RQA Toolbox (v.5.24 (R34) (34). Overall, analysis parameters mirrored those used by Riley and Clark (17), in order to foster comparisons (14), with the exception of modifications to the Theiler Window (as discussed below). The CoP data were first reconstructed in state space using a time delay embedding approach (33) and an iterative process was used to determine each embedding parameter [i.e., embedding dimension (*m*), time delay (*τ*), and recurrence threshold (*r*)]. A false nearest neighbor analysis (47) was performed on each of the CoP signals to determine the embedding dimension (*m*). An embedding dimension (*m*) of 5 was found to yield a reconstruction that maximized the available information. The average displacement method (48) yielded a time delay (*τ*) of 15 samples (i.e., 0.15 s). The recurrence threshold (*r*) was fixed at 5% and was chosen based upon a prior study of RQA and postural control which identified increased reliability with this approach (16).

In the RP, time-contiguous recurrent points forming line segments parallel to the diagonal identity line indicate repeated time series behavior. In accordance with past studies quantifying changes in regularity of the CoP modulated by changes in available sensory information (17), RQA measures included %REC (the percentage of data points identified as recurrent), %DET (the percentage of recurrent points forming line segments parallel to the diagonal identity line in the recurrence plot), and MAXL (the number of points in the longest diagonal line, excluding the main diagonal). Both %DET and %REC are positively related to the predictability or stability of the signal with higher values indicating more predictability (i.e., determinism) and less randomness in the CoP signal (30, 31, 49). MAXL is a measure of dynamical stability inversely proportional to the largest positive Lyapunov exponent (30, 31); thus, shorter MAXL values indicate less mathematically stable (i.e., more chaotic) signals and longer MAXL values indicate increased mathematical stability. While Shannon entropy of the diagonal line structure has previously been investigated, evidence suggests decreased reliability for noisy signals (50), such as that of CoP time series, thus we chose to exclude Shannon entropy from the analysis.

**Figure 2:**
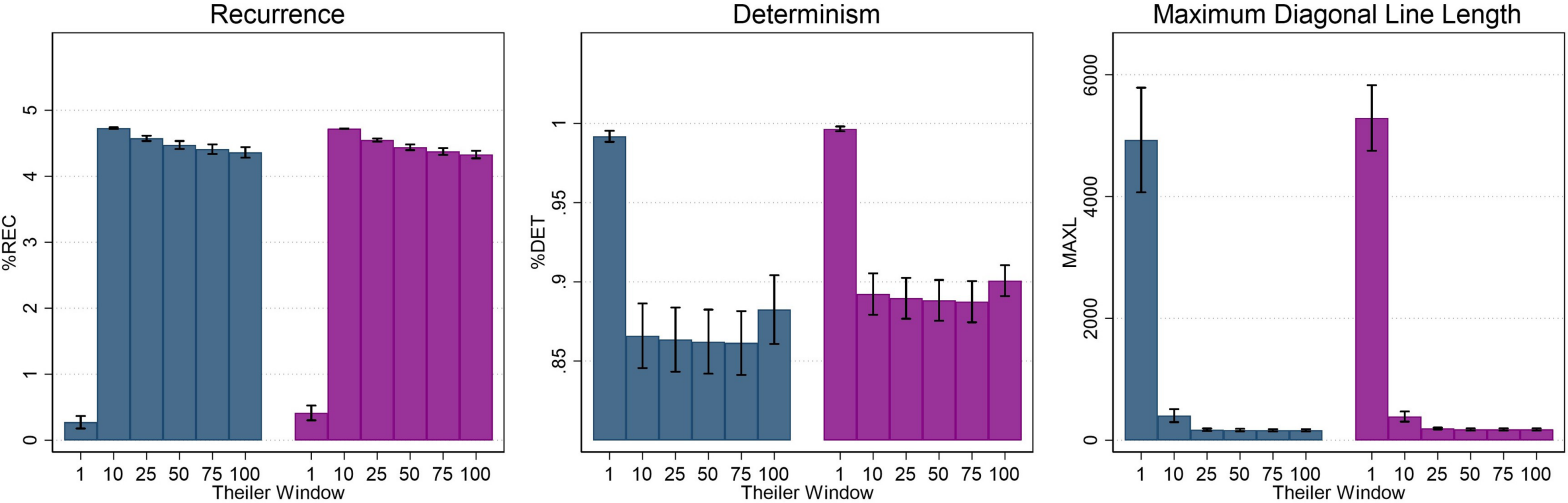
Average recurrence (%REC), determinism (%DET), and maximum diagonal line length (MAXL) as a function of theiler window length (in samples) for condition 1 (firm surface, eyes open). Error bars represent ±0.5 SD.

Use of a Theiler window has also been proposed as a best practice in RQA applications (49). The application of a Theiler window excludes points within a defined boundary surrounding the line of identity (i.e., the main diagonal) and thus eliminates any recurrence that is temporally close. In terms of CoP data, this may preferentially impact larger amplitude motion which is more common in older adults and adults with PPPD (46, 51); inclusion of these large amplitude motions would yield longer diagonal lines and higher determinism values (16, 49). Van den Hoorn et al. (16) proposed using a one second Theiler window for CoP motion, but no studies have empirically determined an appropriate length for implementation. The RQA analyses of the ML CoP in our dataset was repeated for six different Theiler Window lengths (1, 10, 25, 50, 75, and 100 samples). For all five conditions and both participant groups, similar results were seen based on Theiler window alterations. The modulation of non-linear measures on the basis of Theiler window length for Condition 1 is displayed in Figure 2 and Conditions 2–5 can be found in Supplementary Figure S1. For recurrence (%REC), a large and significant increase was noted between 1 and 10 samples (*p* < 0.001); a small but significant decrease (*p* < 0.05) in %REC was noted between 10 and 25 samples, whereas increases beyond 25 did not significantly impact %REC. For determinism, a large and significant decrease was noted with changes in the Theiler Window from 1 to 10 samples (*p* < 0.001); increases beyond 10 samples did not yield a significant change in %DET. Finally, MAXL values significantly decreased as Theiler window increased up to 25 samples, however, values plateaued with additional increases in Theiler window length. As all three metrics (%REC, %DET, and MAXL) plateaued by a Theiler window of 25 samples, a value of 25 was chosen for all remaining analyses.

### 2.3. Statistical analysis

Linear mixed effect models (*mixed*; Stata v. 17.0, College Station, TX) were used to account for the repeated measures design. In the full model, fixed effects of group (HC, PPPD), balance testing condition, interaction of group by condition, and age were included. Separate mixed effects models were used for each linear (i.e., path length, SD) and non-linear outcome measures (i.e., %DET, %REC, MAXL) which included age, group, and balance test condition. As cognitive tasking was only performed while standing with eyes closed on foam, separate analyses for each group were completed to compare this condition to the analogous balance condition without the dual task (i.e., condition 4). Degrees of freedom in all mixed effect models were adjusted using the Kroger method to account for the small sample and unbalanced design (i.e., not all participants were able to complete all balance conditions) (52). *Post hoc* comparisons were completed using tests of simple effects (i.e., partial *F*-tests) to determine the effect of group for each condition of balance testing.

## 3. Results

### 3.1. Linear measures

#### 3.1.1. Path length of the CoP

All HC participants were able to complete all balance testing conditions. However, not all participants diagnosed with PPPD could complete all conditions. One participant with PPPD could not complete conditions 3–5 and an additional participant with PPPD could not complete conditions 4–5 (Table 1).

Overall, a significant effect of age was seen for path length of the ML CoP (*t* = 3.46, *p* = 0.002), while a significant effect of participant group (PPPD vs. HC) was not seen (*F*(1,21.04) = 0.82, *p* = 0.376). A significant impact of condition was seen (*F* = (4,86.29) = 21.26, *p* < 0.001) while a condition by group interaction was not (*F* (4,86.20) = 1.30, *p* = 0.2766).

**Figure 3:**
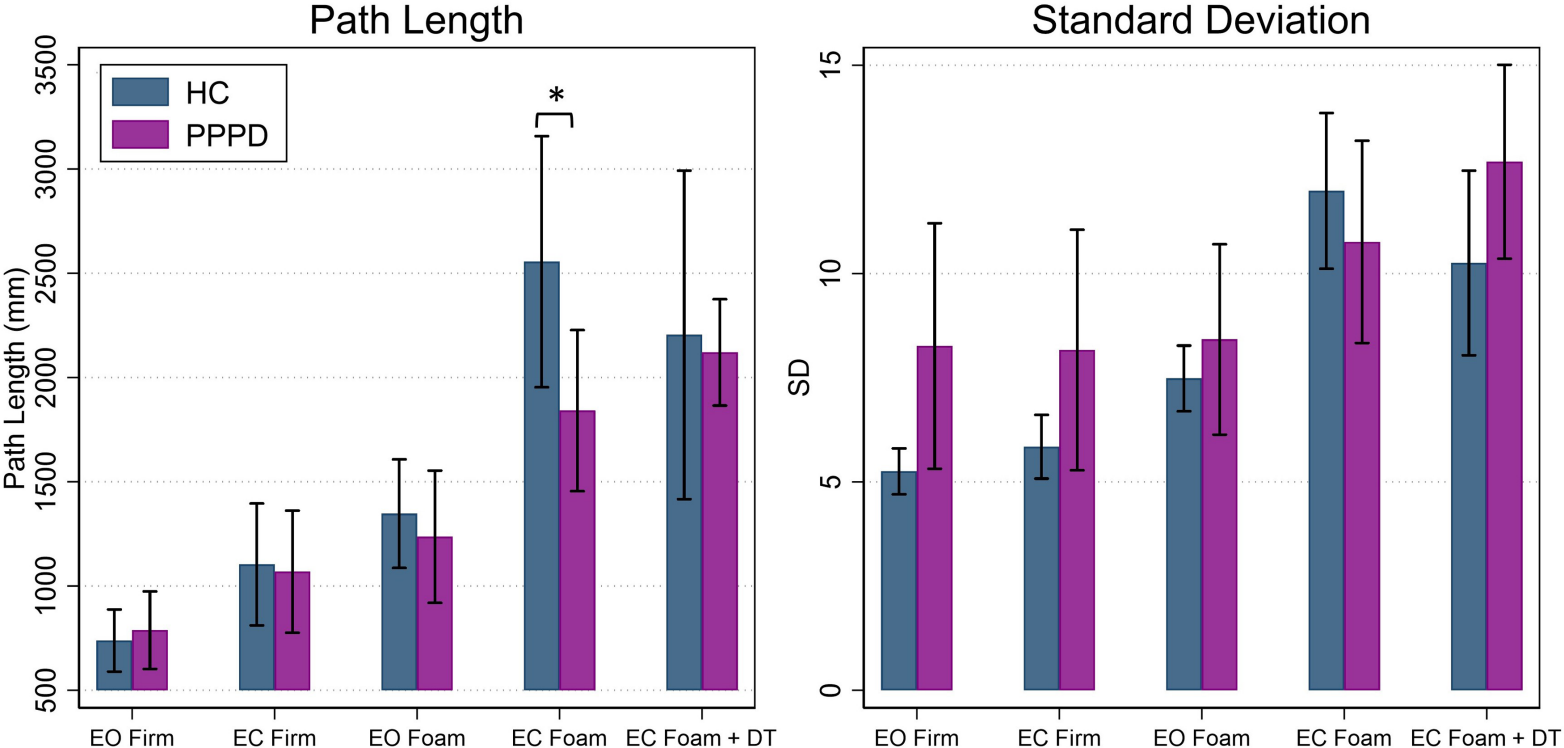
Average path length and standard deviation of the CoP in the mediolateral (ML) plane for healthy controls (navy) and patients with PPPD (purple). Significant pairwise comparisons between groups are marked (*p < 0.05 ). Error bars represent ±0.5 SD. EC, eyes closed; EO, eyes open; DT, dual task.

Table 2 displays statistical results for all *post hoc* testing of linear measures (i.e., path length, standard deviation) comparing performance between groups for each balance condition. Figure 3 displays path length and standard deviation of the CoP for both participant groups. For each condition, a difference between HC and PPPD was only seen for Condition 4, while all other conditions were equivalent between groups. For both HC and PPPD, no effect of additional cognitive task was seen and both Condition 4 and Condition 5 were equivalent (*p* > 0.193).

**Table 2:** Mean and SD of both CoP path length and standard deviation in the medio-lateral (ML) plane.

	HC		PPPD		*F* ratio	*p*
	Mean	SD	Mean	SD		
ML path length						
Main effect	1,589.6	1,156.7	1,368.6	750.0	0.82	0.3767
Eyes open, firm	738.3	298.2	788.0	371.8	0.07	0.7987
Eyes flosed, firm	1,103.4	584.2	1,068.7	585.6	0.01	0.909
Eyes open, foam	1,346.7	520.7	1,236.2	634.2	0.1	0.7508
Eyes closed, foam	2,555.2	1,204.4	1,841.2	772.3	5.54	**0** **.** **0207**
Eyes closed, foam + DT	2,204.6	1,575.6	2,120.4	509.8	0.01	0.9406
ML standard deviation						
Main effect	8.164	3.763	9.501	5.305	1.49	0.2358
Eyes open, firm	5.254	1.100	8.262	5.892	3.72	0.0583
Eyes closed, firm	5.844	1.529	8.166	5.773	1.64	0.2043
Eyes open, foam	7.483	1.577	8.420	4.570	0.34	0.5625
Eyes closed, foam	11.984	3.738	10.762	4.854	0.53	0.4703
Eyes closed, foam + DT	10.256	4.429	12.685	4.655	1.62	0.2067

*F* ratios and *p* values for *post hoc* testing assessing differences between participant groups (i.e., healthy controls vs. adults with PPPD). Significant differences (*p* < 0.05) are in bold. Degrees of freedom were adjusted using the Kroger method to account for the small sample and unbalanced design. DT, dual task; HC, healthy control; PPPD, persistent postural-perceptual dizziness; SD, standard deviation.

#### 3.1.2. Standard deviation of the CoP

For standard deviation of the ML CoP (SD), an overall effect of age (*t* = 1.37, *p* = 0.185) and participant group (*F*(1,21.04) = 1.49, *p* = 0.236) was not identified. There was an overall effect of condition (*F*(4,85.85) = 11.83, *p* < 0.001) but there was not a significant group by condition interaction (*F*(4,85.75) = 1.70, *p* = 0.1578). Additionally, no significant differences were noted between groups for each balance test condition (Table 2; Figure 3). No impact of dual task was seen as Condition 4 and Condition 5 were equivalent for HC (*p* = 0.104) and patients with PPPD (*p* = 0.313).

### 3.2. Non-linear measures

#### 3.2.1. Recurrence

**Figure 4:**
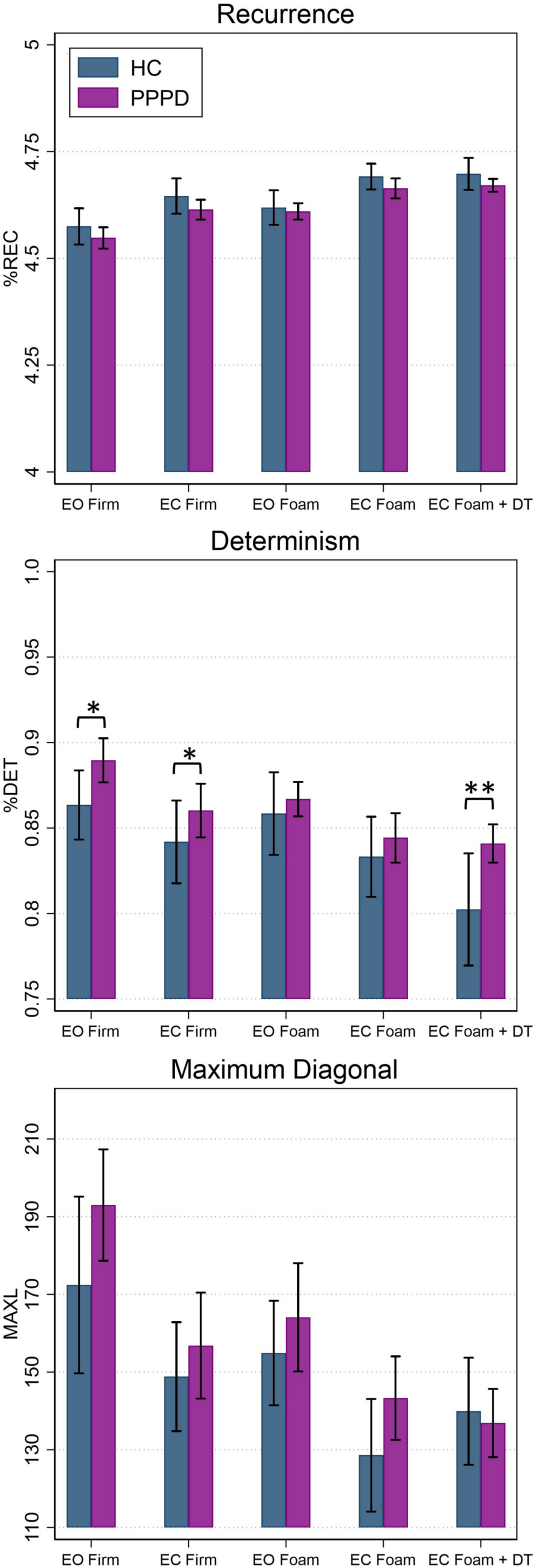
Average percent recurrence (%REC), determinism (%DET), and maximum diagonal length (MAXL) in the mediolateral (ML) plane for healthy controls (blue) and patients with PPPD (purple). Significant pairwise comparisons between groups are marked (*p < 0.05; **p < 0.001). Error bars represent ±0.5 SD. EC, eyes closed; EO, eyes open; DT, dual task.

Figure 4 depicts non-linear measures for both participant groups. Table 3 contains results of statistical analyses comparing HC and individuals with PPPD for all non-linear measures (%REC, %DET, MAXL) for each test condition. %REC demonstrated a significant main effect of age (*t* = 2.36, *p* = 0.029) and condition (*F* (4,82.05) = 34.60, *p* < 0.001). While patients with PPPD trended to exhibit overall higher scores, this effect failed to reach statistical significance (*F*(1,31.75) = 1.32, *p* = 0.109) and there was not a group by condition interaction (*F*(4,82.06) = 0.58, *p* = 0.6779). No significant differences between groups were seen for any conditions (Table 3). No impact of dual task was seen and Condition 4 and Condition 5 were equivalent for both HC (*F*(1,43.27) = 1.23, *p* = 0.203) and PPPD (*F*(1,38.18) = 1.65, *p* = 0.270).

**Table 3:** Mean and SD of percent recurrence (%REC), percent determinism (%DET), and maximum diagonal line length of the CoP in the medio-lateral (ML) plane.

	HC		PPPD		*F* ratio	*p*
	Mean	SD	Mean	SD		
ML recurrence (%REC)						
Main effect	3.883	1.623	3.797	1.680	1.32	0.109
Eyes open, firm	3.849	1.557	3.754	1.581	0.08	0.776
Eyes closed, firm	3.919	1.633	3.794	1.696	0.06	0.803
Eyes open, foam	3.874	1.596	3.798	1.671	0.01	0.939
Eyes closed, foam	3.959	1.676	3.876	1.709	0.05	0.825
Eyes closed, foam + DT	3.808	1.693	3.765	1.823	2.79	0.68
ML determinism (%DET)						
Main effect	0.8651	0.0726	0.8869	0.0580	2.50	**0** **.** **012**
Eyes open, firm	0.8865	0.0589	0.9105	0.0461	5.22	**0** **.** **025**
Eyes closed, firm	0.8669	0.0690	0.8866	0.0577	3.91	**0** **.** **050**
Eyes open, foam	0.8796	0.0648	0.8907	0.0528	1.86	0.176
Eyes closed, foam	0.8577	0.0707	0.8705	0.0621	2.6	0.111
Eyes closed, foam + DT	0.8320	0.0873	0.8704	0.0641	12.22	0.001
ML max diagonal length						
Main effect	153.16	52.17	160.41	31.48	2.82	**0** **.** **049**
Eyes open, firm	171.15	43.79	193.00	28.72	1.72	0.093
Eyes closed, firm	149.69	27.04	156.80	27.28	0.13	0.714
Eyes open, foam	178.23	87.91	164.10	27.86	0.78	0.381
Eyes closed, foam	131.70	29.97	143.30	21.51	0.48	0.492
Eyes closed, foam + DT	133.50	34.41	136.88	17.55	0.63	0.681

*F* ratios and *p* values for *post hoc* testing assessing differences between participant groups (i.e., healthy controls vs. adults with PPPD). Significant differences (*p* < 0.05) are in bold. Degrees of freedom were adjusted using the Kroger method to account for the small sample and unbalanced design. DT, dual task; HC, healthy control; PPPD, persistent postural-perceptual dizziness; SD, standard deviation.

#### 3.2.2. Determinism

For %DET, effects of age (*t* = −2.22, *p* = 0.038), condition (*F*(4,81.19) = 14.21, *p* < 0.001) and participant group (*F*(1,31.57) = 2.50, *p* = 0.0124) were identified. For the participant group effect, average %DET was higher in patients with PPPD. A group by condition interaction was not identified (*F*(4,81.47) = 0.66, *p* = 0.6182). For %DET, a significant difference between HC and PPPD was noted for firm surfaces (Condition 1 & 2) and for dual task performance (Condition 5). An impact of cognitive dual task was identified for HC as Condition 5 was significantly lower than Condition 4 (*F*(1,43.05) = 8.03, *p* = 0.0059); however, no impact was seen for PPPD as Condition 4 and Condition 5 were equivalent (*F*(1,38.73) = 0.78, *p* = 0.3791).

#### 3.2.3. Maximum diagonal line length

MAXL revealed a significant main effect of group (*F*(1,77.08) = 2.82, *p* = 0.0494) as patients with PPPD displayed higher MAXL values. A significant main effect of age (*t* = −1.96, *p* = 0.0648) was not seen. A main effect of condition (*F*(4,81.83) = 5.38, *p* < 0.001) was identified but a condition by diagnosis interaction was not (*F*(4,81.85) = 0.63, *p* = 0.641). A significant difference between participant groups was not noted for any of the individual balance test conditions (Table 3). No impact of dual task was seen as Condition 4 and Condition 5 were equivalent for HC participants (*F*(1,43.29) = 2.10, *p* = 0.154) and patients with PPPD (*F*(1, 35.01) = 0.84, *p* = 0.365).

## 4. Discussion

During quiet stance balance, patients with PPPD and age-matched HC exhibited differences in non-linear measures of postural sway, as quantified by RQA, while linear measures that characterize the magnitude and variability of postural sway did not show systematic differences. These results suggest that patients with PPPD overall exhibit changes in the temporal structure of ML sway, which were not reflected in traditional linear quantification of the CoP signal. A significant difference between HC and PPPD participants was noted for two of the three RQA metrics examined, including %DET, and MAXL, which quantify the diagonal line structures in the recurrence plot and reflect the regularity and predictability of the CoP time series. While %REC trended to be greater in patients with PPPD, this failed to reach statistical significance (*p* = 0.109), potentially reflecting our small sample size in combination with the data variability. Our interpretation of these findings is that patients with PPPD showed a postural sway pattern that was more predictable (i.e., increased %DET and MAXL) than healthy controls. This suggests that patients with PPPD may employ maladaptive compensatory postural control strategies that yield less flexibility in their postural control system. In contrast, the healthy adult control group displayed a more flexible postural control strategy characterized by a lower %DET and shorter maximum line length.

Differences in the deterministic structure of sway between HC and PPPD was shown to be modulated on the basis of balance test condition. For balance conditions on firm surfaces (i.e., Condition 1 and 2), patients with PPPD exhibited increased %DET (i.e., increased regularity of sway) of the ML CoP time series. This suggests that for the less challenging balance tasks, patients with PPPD adopted a more rigid postural control strategy (53). While MAXL demonstrated an overall effect and was significantly higher in patients with PPPD, differences in performance modulated on the basis of task condition were not identified, in part reflecting the heterogeneity in performance. However, a somewhat similar pattern was identified for MAXL as %DET, as the largest difference between groups was seen for Condition 1, which trended to be statistically significant (0.093). Similarities in both the amount and variability of sway (i.e., linear time domain measures) between the two groups suggests that analyzing the underlying structure of sway, rather than only the amount of sway, may serve as a more sensitive technique for describing the changes in postural control that accompany the perceptual symptoms of PPPD. Our data provide further support that patients with PPPD display abnormal postural control strategies, and that such abnormalities are related to the nature of the sensory cues available, and potentially the underlying challenge of the balance task.

Previous applications of RQA to quiet stance balance, have shown an *increase* in the regularity of CoP sway with removal of visual cues and reliable proprioceptive cues in healthy control subjects (14, 17, 20, 22, 23); this is opposite to the behavior we observed in our healthy cohort of older adults, as we instead showed a significant *decrease* in the regularity of sway in the same “eyes closed” conditions. Several methodological differences must however be considered when comparing our data to those of the aforementioned studies. The present study used a longer recording time (60 s vs. 20–30 s), included middle aged and older adults rather than young adults, and constrained the base of support to narrow stance as opposed to a “comfortable width”. Our decision to use a narrow stance posture for balance was intentional, as we intended to challenge medio-lateral postural control (54, 55) due to the relationships between ML sway and fall history (42). The increased challenge relative to comfortable stance may have resulted in the observed decrease in ML CoP regularity under more challenging eyes closed balance tasks in our sample.

Two previous studies assessed non-linear measures of postural control in phobic postural vertigo (PPV) (8, 36), but our study is the first to date to assess similar measures in adults meeting the recently defined diagnostic criteria for PPPD (1). Despite different methodologies and patient diagnoses, our results similarly suggest an overall increase in the regularity and decrease in the complexity of postural control behaviors, with the repetitive nature of CoP sway in patients with PPPD being characteristic of a more rigid and less adaptable postural control system (53).

In a sample of 12 patients with PPV, Schniepp et al. (56) identified a decrease in the ML and AP sample entropy, indicating increased regularity, and a lower scaling exponent, indicating increased strength of long-range correlations in the CoP signal, during quiet stance with the eyes open and closed, while standing on either firm or foam surfaces. With increasing demands of the balance task (i.e., eyes closed on foam), PPV patients showed normalization of entropy values, and a similar level of complexity in the CoP signal to healthy controls (36).

Similarly, in patietns with PPV, Wuehr et al. (8) identified a higher scaling exponent and higher short-term diffusion coefficients during eyes open and eyes closed stance on a firm surface, which were less prominent during the more challenging balance task (i.e., when standing eyes closed). These results are in line with the hypothesis that patients with PPV use a postural control strategy that is typically employed only for demanding balance tasks. Overall, these results are consistent with our identified increases in %DET and MAXL across condition for patients with PPPD. As well, we found a significantly higher recurrence rate for condition 1 (eyes open, firm) which normalized with increases in task difficulty, suggesting that patients with PPPD exhibit changes in the dynamic structure of postural control even for less challenging balance tasks.

In contrast to some past findings (6, 8, 9, 12, 46), we failed to identify increases in traditional, linear measures of balance performance (path length and SD) during quiet stance in patients with PPPD. This may reflect our smaller sample size (*n* = 12) or potential subgroups in patients with PPPD. Past studies that have focused upon quantifying the amount of sway, as quantified by root mean square displacement (RMSD), in PPV relative to healthy controls, have shown an overall increase in sway during assessments of balance performed with the eyes open/eyes closed on a firm surface (11) and eyes open/eyes closed in normal or tandem stance on a firm, as well as foam surface (12). However, patients with PPV or PPPD have also been found to demonstrate improved balance performance (i.e., decreases in RMS distance or decreases in degree of trunk sway) relative to healthy controls for more difficult balance tasks (12, 56). We did not explicitly compare postural sway between each participant group for each balance test condition, however, both HC and PPPD exhibited an increase in sway (i.e., greater path length) as task difficulty increased. HC participants also displayed a characteristic increase in the variability of sway (i.e., increased SD) which mirrored the increases in path length. In patients with PPPD, the SD was instead similar for Condition 1 (eyes open/firm), Condition 2 (eyes closed/firm), and Condition 3 (eyes open/foam), despite coexistent increases in the amount of sway. These data suggest that patients with PPPD may display increased variability in the CoP for both “easy”, as well as “challenging” balance tasks. Increases in sway, using the sensory organization test (SOT), have previously been reported in patients with PPPD (6, 46). However, the predominant differences observed in these studies between PPPD and asymptomatic controls was found in conditions 2–6, while sway assessed with eyes open on a fixed surface was not significantly impacted in patients with PPPD (6, 46). Of note, traditional posturography manipulates availability of proprioceptive and visual cues through either sway referencing (i.e., moving the visual scene or platform in concert with an estimate of body sway) of the support surface and visual surround in the anterior-posterior (AP) plane, or through the removal of visual inputs (eyes closed stance) (58). The use of a sway referenced visual scene, as opposed to the removal of visual inputs, allows for the characterization of an individual's dependence upon visual cues, as “visually dependent” persons will continue to utilize the erroneous cues despite increases in postural sway (59, 60). As patients with PPPD have been reported to display visual dependency (61, 62), the use of unreliable visual feedback, rather than the complete removal of visual cues as was done in the present study, may have better captured the postural control strategies of patients with PPPD.

Past studies have reported changes in postural sway patterns which may vary across PPPD or CSD patients (56, 63). Potential subtypes of PPPD have been proposed on the basis of symptomology (64) with a portion of patients displaying more pronounced balance impairments. In patients with PPPD, a subset display both dizziness and impaired balance performance during standardized gait and stance tasks while others display dizziness only (56). Similarly, in patients with CSD, a subset of patients display unremarkable or narrower sway paths, which is not captured on standard scoring of the SOT, while others may display overtly abnormal postural sway patterns (63). Use of RQA or other non-linear analysis of postural sway patterns may provide insights into these potential subgroups of PPPD patients.

In healthy adults, the addition of a cognitive dual task challenge to a balance assessment has been consistently shown to impact the temporal structure of CoP, leading to a decrease in the regularity (i.e., decreased determinism and recurrence) of sway (23, 26, 65). However, these modulations were shown to be dependent on task difficulty, as in general, larger decreases were noted for the more challenging dual task conditions (23, 65). In our cohort of healthy adults, we similarly noted a decrease in MAXL and %DET in the dual task condition, without a concurrent change in the amount of sway or in the variability of sway. However, in PPPD, a significant impact of dual task was only seen for %DET and was not observed for any of the other linear or non-linear metrics. Past reports suggested that in PPV, the addition of a dual task challenge during stance on a firm surface, with either the eyes open or closed, led to improvements in RMSD and SDA metrics (i.e., short term diffusion and critical time interval) yielding a normalization of performance relative to healthy adults (11). However, similar findings were not identified in our current investigation that included a dual task challenge during the “eyes closed, on foam” condition. In patients with PPPD, no significant differences were noted for any linear or non-linear metrics when comparing performance in the dual task condition to performance during the same task without the added cognitive load. Our methodology did however differ from the past study by Wuehr et al. (11) as we employed a cognitive dual task challenge during a condition which was more challenging and where balance performance was expected to rely more heavily upon vestibular cues (i.e., secondary to the removal of reliable visual and proprioceptive cues). A different cognitive task was also used in the previous study (i.e., naming from a category) than the current investigation (i.e., counting backwards by threes). Future investigations should further investigate the impact of a distracting cognitive task on balance performance in PPPD by administering dual task challenges in various balance conditions (e.g., eyes open, eyes closed, firm, and foam surfaces) in order to determine the potential interaction between cognitive demand and the availability of sensory information.

Although our conclusions are straight forward, our analysis and interpretation is limited by a somewhat small sample size. We included a large age range within both groups. While we age-matched between groups and adjusted for age within our statistical modeling, there may have been an impact of age that we were unable to identify. The distribution of males and females was not equal, but both the sex and age distributions of patients with PPPD included in this investigation are in line with other reports of PPPD patient demographics (4, 66, 67). However, PPPD with coexisting vestibular migraine represented a slightly higher proportion (∼50%) of patients in comparison to other studies of PPPD patient population (∼30%) (4). As our primary recruitment source was an oto-neurology practice, this likely reflects the differences in patient populations between this specialty clinic and other tertiary care centers. As well, the aim of this study was to investigate the utility of RQA in order to inform future efforts investigating changes in the dynamics of the temporal structure of CoP sway in patients with PPPD. We were able to use RQA to identify differences in dynamics of postural control between a group of patients with PPPD in comparison to a group of age-matched healthy controls. The differences suggest that postural sway complexity and regularity is modulated in PPPD and that patients with PPPD may exhibit maladaptive postural control behaviors even during non-challenging balance demands. These group differences were not noted for traditional linear measures, suggesting that RQA and other metrics may provide unique insights into postural control mechanisms and could potentially serve as a biomarker for diagnosis.

Future efforts should quantify if changes in postural control strategies as quantified by RQA may occur in response to therapeutical interventions (e.g., medications, vestibular rehabilitation therapy). However, three patients were currently enrolled in vestibular rehabilitation, but it is unknown whether or not other participants had completed therapy at outside practices which may have impacted balance performance. All PPPD participants still reported actively experiencing PPPD symptoms, but future studies should examine rehabilitation as a potential influence. As well, future endeavors should enroll a larger sample of participants to provide statistical power to expand the focus of the present study, including separate analyses to investigate the effect of PPPD on other dimensions of postural sway. As anxiety and neuroticism have been proposed to play an role in the progression of PPPD symptoms and the processing of visual motion stimuli (10, 68), future investigations should also assess the correlative relationship between RQA metrics and self-report measures of symptoms, including state and trait anxiety.

## Data Availability

The raw data supporting the conclusions of this article will be made available by the authors, without undue reservation.
