# Supplementary material for: Recurrence quantification analysis of postural sway in patients with persistent postural perceptual dizziness
Source: Front Rehabil Sci. 2023 Jul 27;4:1142018. doi: 10.3389/fresc.2023.1142018 (PMC10415033; doi:10.3389/fresc.2023.1142018)
Supplement: Supplementary file 1 [file Datasheet1.docx]

Supplementary Material

**Recurrence Quantification Analysis of Postural Sway in Patients with Persistent Postural Perceptual Dizziness**

**Megan J. Kobel^*^, Andrew R. Wagner, and Daniel M. Merfeld**

*** Correspondence:** Megan Kobel, AuD, PhD: [kobel.6@osu.edu](mailto:kobel.6@osu.edu)

|  | **HC** | **PPPD** |
| --- | --- | --- |
| **Gender** | 7F/5M | 11F/1M |
| **Age** | 46.5 (12.5) | 45.1 (12.9) |
| **BAI** | 0 (0-19) | 31.2 (18-50) |
| **PHQ-9** | 0 (0-12) | 12.7 (5-21) |
| **DHI** | 0 (0-4) | 65.0 (38-88) |
| **NPQ** | 0 (0-4) | 36.4 (14-54) |
| **VVAS** | 0.7 (0-4.5) | 5.5 (0.5-8.7) |
| **ABC** | 93.5 (92.3-99.3) | 46.25 (26.25-81.25) |
| **BAI** | 1 (0-28) | 31.2 (18-50) |
| **PHQ-9** | 0 (0-12) | 12.7 (5-21) |

**Supplemental Table 1.** Demographic information for participant groups including mean age and standard deviation. Median and minimum and maximum scores for standardized questionnaires are presented as scores were not normally distributed. All patients with PPPD exhibited moderate to severe anxiety and mild to severe depression symptoms. Two HC participants reported anxiety and one HC participant reported moderate depression. These HC participants exhibited linear and non-linear balance metrics which were within one SD of the mean. All PPPD patients reported presence of PPPD symptoms, as indicated by the NPQ, and a moderate to severe dizziness handicap. All HC participants denied significant dizziness or balance deficits. HC = healthy control; PPPD = persistent postural-perceptual dizziness; DHI = Dizziness Handicap Inventory (range 0-100); NPQ = Niigata PPPD Questionnaire (range 0- 72); VVAS = Visual Vertigo Analogue Scale (range 0-10); ABC = Activities Balance Confidence Scale (range 0-100); BAI = Beck Anxiety Inventory (range 0-63); PHQ- 9 = Patient Health Questionnaire – 9 (range 0-27).

|  | **HC** | | **PPPD** | | **F ratio** | ***p*** |
| --- | --- | --- | --- | --- | --- | --- |
|  | **Mean** | **SD** | **Mean** | **SD** |  |  |
| **AP Path length** | | | | | | |
| Main Effect | 1848.6 | 1740.27 | 1460.2 | 815.46 | 1.22 | 0.3085 |
| Eyes Open, Firm | 730.0 | 494.2 | 933.9 | 560.6 | 0.32 | 0.5759 |
| Eyes Closed, Firm | 1274.2 | 1206.6 | 1063.4 | 558.9 | 0.21 | 0.6494 |
| Eyes Open, Foam | 1357.7 | 731.7 | 1226.6 | 555.2 | 0.06 | 0.8043 |
| Eyes Closed, Foam | 2917.3 | 1849.4 | 2009.9 | 831.5 | **4.34** | **0.0399** |
| Eyes Closed, Foam + DT | 2963.7 | 2449.0 | 2283.8 | 673.4 | 1.39 | 0.2411 |
| **AP Standard Deviation** | | | | | | |
| Main Effect | 8.7207 | 3.979 | 10.387 | 5.3956 | 1.95 | 0.0954 |
| Eyes Open, Firm | 5.4586 | 1.4203 | 9.8974 | 6.4748 | **7.55** | **0.0073** |
| Eyes Closed, Firm | 5.9913 | 2.0318 | 8.2393 | 4.8292 | 1.51 | 0.2225 |
| Eyes Open, Foam | 8.5677 | 1.9469 | 9.9142 | 5.6112 | 0.67 | 0.4153 |
| Eyes Closed, Foam | 12.0971 | 3.3162 | 11.215 | 4.8258 | 0.23 | 0.6296 |
| Eyes Closed, Foam + DT | 11.4886 | 4.8404 | 13.2458 | 4.3754 | 1.08 | 0.301 |

**Supplemental Table 2.** Mean and SD of both COP path length and Standard Deviation of the CoP in the anterior-posterior (AP) plane. F ratios and p values for post-hoc testing assessing differences between participant groups (i.e., healthy controls vs. adults with PPPD) for linear measures of CoP. Significant differences (*p* < 0.05) are in bold. Degrees of freedom were adjusted using the Kroger method to account for the small sample and unbalanced design. DT = dual task, HC = healthy control, PPPD = persistent postural-perceptual dizziness, SD = standard deviation.

|  | **HC** | | **PPPD** | | **F ratio** | ***p*** |
| --- | --- | --- | --- | --- | --- | --- |
|  | **Mean** | **SD** | **Mean** | **SD** |  |  |
| **AP Recurrence (%REC)** | | | | | | |
| Main Effect | 4.627 | 0.265 | 4.623 | 0.070 | 0.29 | 0.590 |
| Eyes Open, Firm | 4.611 | 0.066 | 4.567 | 0.080 | 0.35 | 0.565 |
| Eyes Closed, Firm | 4.656 | 0.051 | 4.613 | 0.066 | 0.23 | 0.629 |
| Eyes Open, Foam | 4.659 | 0.043 | 4.632 | 0.063 | 0.09 | 0.767 |
| Eyes Closed, Foam | 4.687 | 0.041 | 4.658 | 0.042 | 0.11 | 0.745 |
| Eyes Closed, Foam + DT | 4.511 | 0.613 | 4.664 | 0.042 | 3.17 | 0.075 |
| **AP Determinism (%DET)** | | | | | | |
| Main Effect | 0.810 | 0.088 | 0.815 | 0.051 | 1.01 | 0.319 |
| Eyes Open, Firm | 0.810 | 0.036 | 0.812 | 0.070 | 1.01 | 0.319 |
| Eyes Closed, Firm | 0.805 | 0.035 | 0.807 | 0.071 | 1.11 | 0.295 |
| Eyes Open, Foam | 0.807 | 0.029 | 0.819 | 0.036 | 1.06 | 0.306 |
| Eyes Closed, Foam | 0.818 | 0.035 | 0.818 | 0.036 | 0.90 | 0.345 |
| Eyes Closed, Foam + DT | 0.813 | 0.187 | 0.817 | 0.034 | **10.09** | **0.002** |
| **AP Max Diagonal Length** | | | | | | |
| Main Effect | 145.8 | 29.8 | 168.9 | 44.1 | **3.75** | **0.040** |
| Eyes Open, Firm | 170.1 | 32.3 | 184.3 | 70.1 | 1.03 | 0.312 |
| Eyes Closed, Firm | 147.3 | 26.2 | 156.3 | 29.3 | 0.31 | 0.580 |
| Eyes Open, Foam | 139.3 | 22.3 | 160.0 | 35.3 | 1.94 | 0.167 |
| Eyes Closed, Foam | 139.8 | 21.4 | 137.4 | 21.4 | 0.02 | 0.888 |
| Eyes Closed, Foam + DT | 131.4 | 34.1 | 150.0 | 24.7 | 1.39 | 0.242 |

**Supplemental Table 3.** Mean and SD of percent recurrence, percent determinism, and maximum diagonal line length of the COP in the anterior-posterior (AP) plane. F ratios and p values for post-hoc testing assessing differences between participant groups (i.e., healthy controls vs. adults with PPPD) for linear measures of CoP. Significant differences (*p* < 0.05) are in bold. Degrees of freedom were adjusted using the Kroger method to account for the small sample and unbalanced design. DT = dual task, HC = healthy control, PPPD = persistent postural-perceptual dizziness, SD = standard deviation.

**
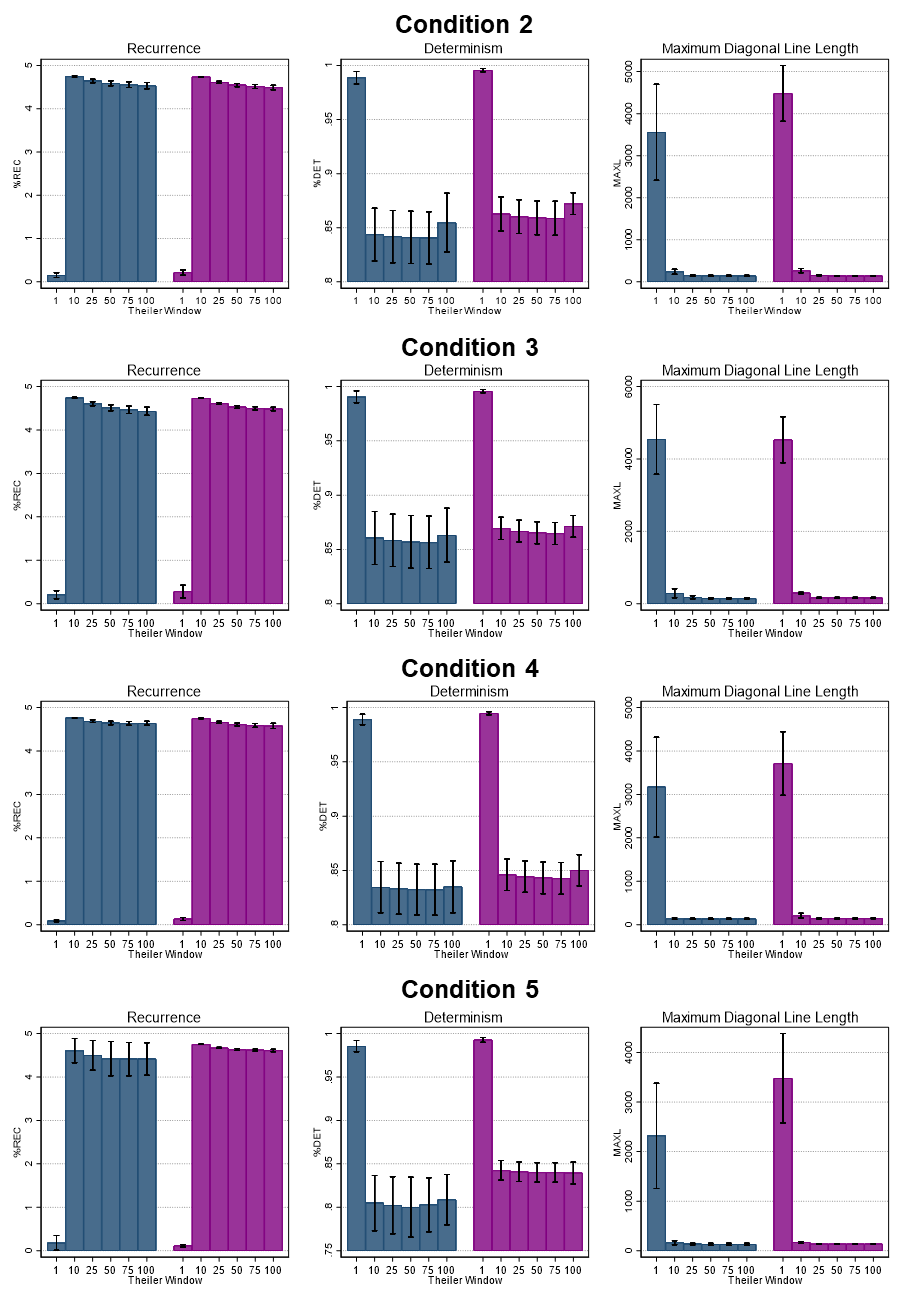
**

**Supplemental Figure 1.** Average recurrence (%REC), determinism (%DET), and maximum diagonal line length (MAXL) as a function of Theiler window length for Condition 2 (eyes closed, firm surface), Condition 3 (eyes open, foam surface), Condition 4 (eyes closed, foam surface), and Condition 5 (eyes closed, foam surface + dual task). Error bars represent +/-1 SD. Statistical comparisons between metrics for Theiler window lengths were completed for each condition using mixed effect linear models with fixed effects including Theiler window length, participant group (HC vs. PPPD), and participant age. Degrees of freedom were adjusted using the Kroger method (Kenward and Roger, 1997) to account for the small sample size and unbalanced design, as not all participants were able to complete all balance conditions. Post-hoc comparisons were completed using partial F-tests and a Bonferroni correction was utilized for multiple comparisons. Post-hoc comparisons are not graphically depicted due to the number of comparisons performed. Full results are summarized in body of the text. In brief, %REC was significantly smaller for a 1 and 10 sample Theiler window in comparison to 25 samples and longer (p<0.05). Percent determinism (%DET) was significantly larger for 1 sample in comparison to all other Theiler window lengths (p<0.001). Max line length (MAXL) was significantly longer for 1 and 10 sample Theiler window lengths in comparison to all other window lengths.
